# Evaluating Organism-Wide Changes in the Metabolome and Microbiome following a Single Dose of Antibiotic

**DOI:** 10.1128/mSystems.00340-20

**Published:** 2020-10-06

**Authors:** Alison Vrbanac, Kathryn A. Patras, Alan K. Jarmusch, Robert H. Mills, Samuel R. Shing, Robert A. Quinn, Fernando Vargas, David J. Gonzalez, Pieter C. Dorrestein, Rob Knight, Victor Nizet

**Affiliations:** a Department of Pediatrics, UC San Diego, La Jolla, California, USA; b Department of Molecular Virology and Microbiology, Baylor College of Medicine, Houston, Texas, USA; c Skaggs School of Pharmacy and Pharmaceutical Sciences, UC San Diego, La Jolla, California, USA; d Department of Pharmacology, UC San Diego, La Jolla, California, USA; e Department of Biochemistry and Molecular Biology, Michigan State University, East Lansing, Michigan, USA; f Center for Microbiome Innovation, UC San Diego, La Jolla, California, USA; g Department of Computer Science and Engineering, UC San Diego, La Jolla, California, USA; h Department of Bioengineering, UC San Diego, La Jolla, California, USA; Vanderbilt University Medical Center

**Keywords:** 3D data visualization, antibiotics, mass spectrometry, metabolome, microbiome

## Abstract

Antibiotics are a mainstay of modern medicine, but as they kill their target pathogen(s), they often affect the commensal microbiota. Antibiotic-induced microbiome dysbiosis is a growing research focus and health concern, often assessed via analysis of fecal samples. However, such analysis does not inform how antibiotics influence the microbiome across the whole host or how such changes subsequently alter host chemistry. In this study, we investigated the acute (1 day postadministration) and delayed (6 days postadministration) effects of a single parenteral dose of two common antibiotics, ampicillin or vancomycin, on the global metabolome and microbiome of mice across 77 different body sites from 25 different organs. The broader-spectrum agent ampicillin had the greatest impact on the microbiota in the lower gastrointestinal tract (cecum and colon), where microbial diversity is highest. In the metabolome, the greatest effects were seen 1 day posttreatment, and changes in metabolite abundances were not confined to the gut. The local abundance of ampicillin and its metabolites correlated with increased metabolome effect size and a loss of alpha diversity versus control mice. Additionally, small peptides were elevated in the lower gastrointestinal tract of mice 1 day after antibiotic treatment. While a single parenteral dose of antibiotic did not drastically alter the microbiome, nevertheless, changes in the metabolome were observed both within and outside the gut. This study provides a framework for how whole-organism -omics approaches can be employed to understand the impact of antibiotics on the entire host.

**IMPORTANCE** We are just beginning to understand the unintended effects of antibiotics on our microbiomes and health. In this study, we aimed to define an approach by which one could obtain a comprehensive picture of (i) how antibiotics spatiotemporally impact commensal microbes throughout the gut and (ii) how these changes influence host chemistry throughout the body. We found that just a single dose of antibiotic altered host chemistry in a variety of organs and that microbiome alterations were not uniform throughout the gut. As technological advances increase the feasibility of whole-organism studies, we argue that using these approaches can provide further insight on both the wide-ranging effects of antibiotics on health and how to restore microbial communities to mitigate these effects.

## Abstract (precis)

We are just beginning to understand the unintended effects of antibiotics on our microbiomes and health. In this study, we aimed to define an approach by which one could obtain a comprehensive picture of (i) how antibiotics spatiotemporally impact commensal microbes throughout the gut and (ii) how these changes influence host chemistry throughout the body. We found that just a single dose of antibiotic altered host chemistry in a variety of organs and that microbiome alterations were not uniform throughout the gut. As technological advances increase the feasibility of whole-organism studies, we argue that using these approaches can provide further insight on both the wide-ranging effects of antibiotics on health and how to restore microbial communities to mitigate these effects.

## OBSERVATION

Antibiotics, a cornerstone of modern medicine, have saved countless lives from infectious disease since their widespread introduction in the 1940s (1). However, in recent years, the collateral damage that antibiotics inflict on the commensal microbiome has become an increasing health concern. By ablating commensal microbes, antibiotics can allow secondary infections, such as Clostridioides difficile colitis (2) and candidiasis (3). Early life antibiotic exposure is particularly damaging (4) and has been associated with greater risk of asthma (5), eczema (6), inflammatory bowel disease (7), and obesity (8).

For antibiotic-induced microbiome perturbation to be transduced into altered disease risk, changes in the metabolism and/or molecular composition of the host must occur. However, little is known about how antibiotic-induced microbiome changes alter the overall chemical makeup of the body. Numerous studies have used fecal samples to assess microbiome composition (9–11) and stool metabolites (12–14) after antibiotic treatment. Here, we deployed novel mass spectrometry informatics and three-dimensional whole-organism data visualization approaches (15) to understand how a single dose of parenteral antibiotics affects the gastrointestinal (GI) tract microbiome and the local metabolome of every organ. We treated 10 mice with commonly prescribed parenteral antibiotics, ampicillin (AMP), a broad-spectrum beta-lactam, and vancomycin (VAN), a narrower-spectrum glycopeptide with activity against Gram-positive bacteria (16), and 10 mice were treated with phosphate-buffered saline (PBS; control) by intraperitoneal (i.p.) injection. One day and 6 days later, we dissected mice to sample 77 different body sites from 25 different organs to evaluate the whole-organism impact of antibiotics acutely and after microbiome recovery. We analyzed all samples by liquid chromatography-tandem mass spectrometry (LC-MS/MS) and the gastrointestinal tract samples (*n* = 38 per mouse) by 16S rRNA sequencing (see Fig. S1 in the supplemental material).

10.1128/mSystems.00340-20.1 FIG S1 Overview of study design. Thirty-seven-week-old mice were ordered from Jackson Laboratories and randomly housed 2 or 3 mice per cage. After a 6-day rest period, mice were administered either 100 μl PBS, 100 mg/kg AMP in 100 μl PBS, or 100 mg/kg VAN in 100 μl PBS via intraperitoneal injection. Twenty-four hours later, 5 mice per group were dissected by following the protocol detailed in the methods. Six days postantibiotic administration, the remaining 5 mice per group were dissected by following the protocol detailed in the methods. Download FIG S1, TIF file, 1.4 MB. Copyright © 2020 Vrbanac et al. 2020 Vrbanac et al. This content is distributed under the terms of the Creative Commons Attribution 4.0 International license.

### Antibiotic impacts on the microbiome.

The greatest effects on the microbiome occurred in mice that had received antibiotics 24 h prior (AMP.d1 and VAN.d1). Compared to control mice, the centered log ratio (clr)-transformed proportion of Gram-positive bacteria was significantly reduced in the fecal pellet, certain sections of the stomach and colon of AMP.d1 and VAN.d1 mice, and certain sections of the cecum in AMP.d1 mice (Fig. S2).

10.1128/mSystems.00340-20.2 FIG S2 Antibiotics reduce levels of Gram-positive bacteria. Asterisks indicate a significant difference in the centered log ratio (clr)-transformed proportion of Gram-positive bacteria compared to the control (Mann-Whitney U test, *P* < 0.05 after Benjamini-Hochberg correction for multiple comparisons). Error bars represent the 95% confidence interval (CI). Download FIG S2, TIF file, 1.1 MB. Copyright © 2020 Vrbanac et al. 2020 Vrbanac et al. This content is distributed under the terms of the Creative Commons Attribution 4.0 International license.

**FIG 1:**
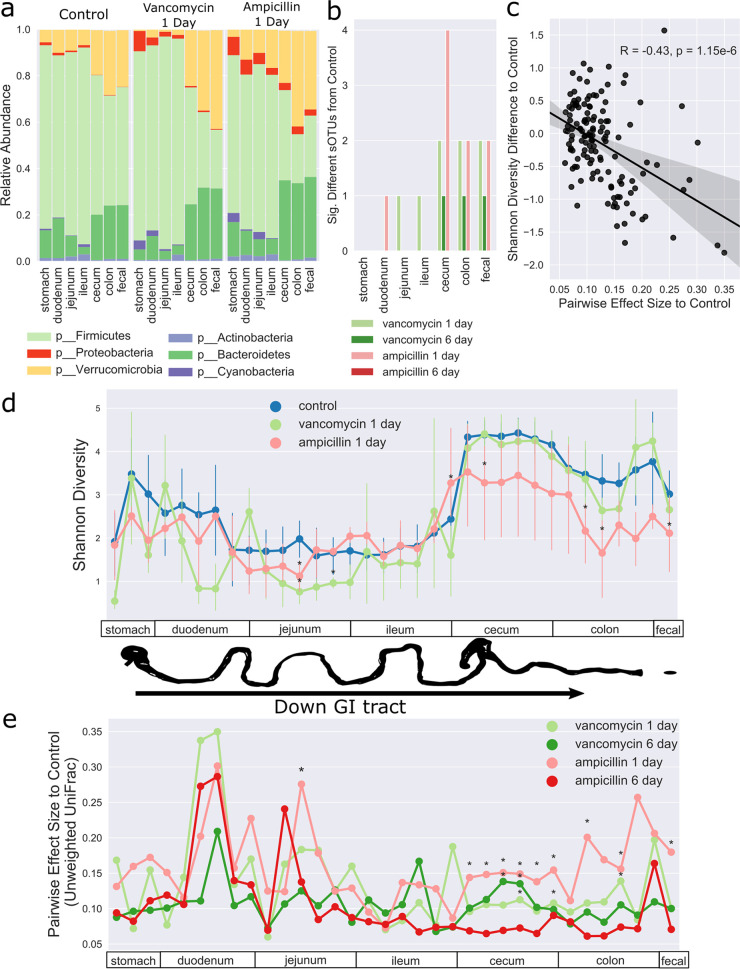
Antibiotics affect the GI microbiome. (a) Stacked bar plots of the average relative abundance of bacterial phyla by organ in control mice and in mice 1 day after antibiotic treatment. (b) Count plot of significantly different sOTUs compared to the control; significance testing was performed with ANCOM (44). (c) Spearman correlation of changes in Shannon diversity from the control with pairwise effect size (e) for every noncontrol sample; the shaded area represents the 95% confidence interval (CI). (d) Shannon diversity down the GI tract. Asterisks indicate Shannon diversity significantly different from the control (Mann-Whitney U test with a Benjamini-Hochberg FDR control level of 0.1); error bars represent the 95% CI. (e) Pairwise effect size for each group compared to control samples, progressing down the GI tract. Significance was determined by PERMANOVA with a Benjamini-Hochberg FDR control level of 0.1 (48). The effect size is the PERMANOVA *R*^2^ value.

Compositionally, an increase in proteobacteria at the phylum level was observed in both AMP.d1 (cecum and fecal) and VAN.d1 mice (duodenum and stomach), differentially abundant sub-operational taxonomic units (sOTUs), also known as amplicon sequence variants or exact sequence variants, were seen in both AMP.d1 and VAN.d1 mice, and the level of one *Clostridium* sOTU was significantly lower in VAN.d6 mice (Fig. 1a and b). Antibiotic treatment also reduced microbiome alpha diversity (Fig. 1d). Specifically, Shannon diversity was reduced in the lower GI tract (cecum, colon, and fecal pellet) of AMP.d1 mice and in the upper GI tract (jejunum) of VAN.d1 and AMP.d1 mice. We next calculated unweighted UniFrac distance (17) for each group and compared effect size to those of controls down the GI tract (Fig. 1e). AMP.d1 mice had significantly altered beta diversity with high effect sizes, particularly in the lower GI tract, while beta diversity changes for VAN.d1 mice were only significant in a few GI sections. Effect size and diminished Shannon diversity were significantly correlated (Fig. 1c). AMP had a greater effect on the gut microbiota overall than VAN, consistent with its broader spectrum, recognizing that the biodistribution of parenteral VAN into the gut is poorly studied. Both AMP and VAN are renally excreted, and it is possible that some antibiotic was consumed orally via coprophagy through urine contacting fecal pellets.

### The global metabolome.

**FIG 2:**
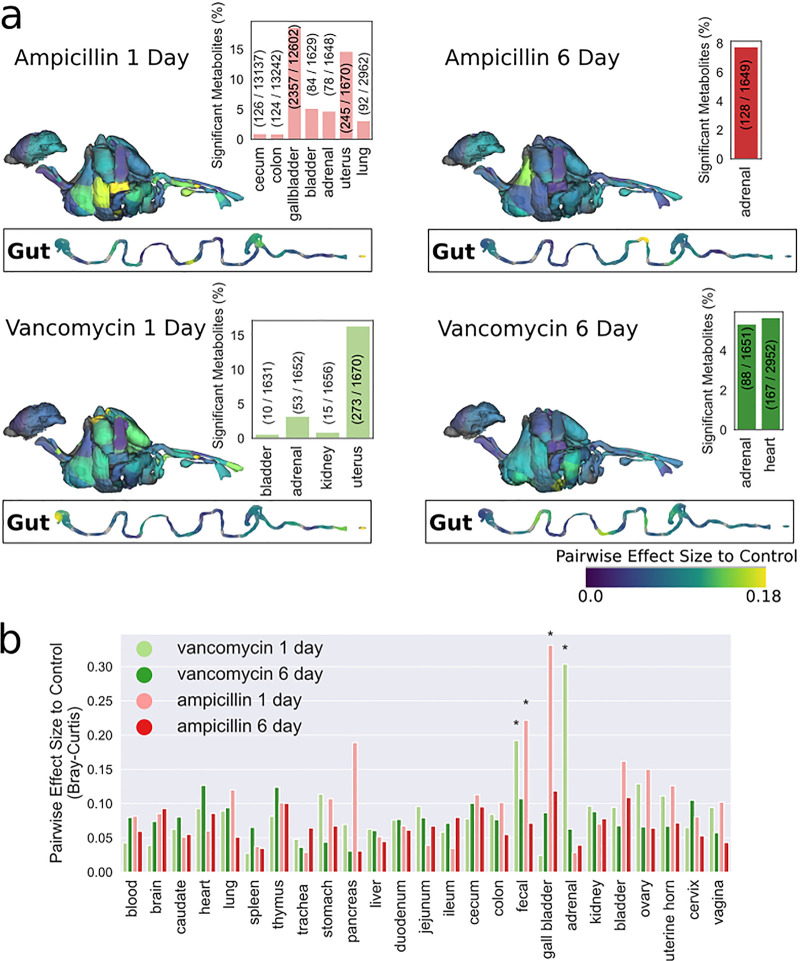
Organism-wide impact of antibiotics on the metabolome. (a) Pairwise effect size for each group compared to control samples, mapped onto body site using a 3D mouse model and a 2D illustration of the GI tract and percentage of significantly different metabolites compared to the control; significance was determined by dsFDR (47) (FDR control level, 0.1). (b) Pairwise effect size for each group compared to control samples. For body sites with multiple samples (i.e., colon cut into 6 sections), the metabolites were averaged for each mouse prior to calculating Bray-Curtis distance. Significance was determined by PERMANOVA with a Benjamini-Hochberg FDR control level of 0.1 (48), and effect size is the PERMANOVA *R*^2^ value.

To evaluate the global impact of antibiotics on metabolome composition, we calculated effect size using Bray-Curtis distance comparing each treatment group to controls and mapped the effect-size data onto a three-dimensional (3D) mouse model to provide a spatial visualization (Fig. 2a). For each body site/organ, averaging those with multiple samples (e.g., colon with 6 sections), only AMP.d1 and VAN.d1 showed significant differences in beta diversity (Fig. 2b). Both AMP.d1 and VAN.d1 had significantly different Bray-Curtis distance versus control mice in fecal samples. In peripheral organs, AMP.d1 mice differed significantly from the control in Bray-Curtis distance in the gallbladder, while VAN.d1 mice differed significantly in adrenal glands. We observed differentially abundant features in various organs compared to control mice (Fig. 2a, inset barplots), some putatively annotated with Global Natural Product Social Molecular Networking (GNPS) (18). Gallbladders of AMP.d1 mice had many differentially abundant features, including elevated bile acids (cholic, taurocholic, taurodeoxycholic, and cholenic acids). Uterine tissue of VAN.d1 and AMP.d1 mice had significantly higher levels of arachidonic acid, its metabolites, including 8-HETE and prostaglandin E2, and linoleic acid metabolites, such as 9-OxoODE. Significantly different features in adrenal glands of all antibiotic treatment groups compared to the control included a variety of elevated acylcarnitines and oxidized glutathione (GSSG). Glutathione is a key antioxidant in the adrenal cortex, where steroidogenesis generates reactive oxygen species (19), and GSSG is a biomarker of oxidative stress (20). VAN is nephrotoxic (21), and only kidneys of VAN.d1 mice had differentially abundant features, including elevated palmitoylcarnitine, a potential marker of renal toxicity in rats (22).

Molecular networking analysis (18) of the raw metabolomics data revealed a cluster of features (molecules or metabolites) associated with AMP (Fig. S3). Many of these network features were present in our MS1 feature table and only in AMP.d1 mice, so these were summed to examine the collective distribution of AMP and its metabolites. Concentrated in the lower GI tract of AMP.d1 mice (Fig. S4A), the abundance of these AMP network features correlated with metabolome effect size (Bray-Curtis distance) and reduced Shannon diversity versus control mice (Fig. S3C and S4B), indicating that metabolome and microbiome effects were greatest in areas of high local AMP concentration. VAN (molecular weight, 1,449.2 g/mol) was not detected in our samples, limited by the LC-MS/MS MS1 scan range of *m/z* 100 to 1,500.

10.1128/mSystems.00340-20.3 FIG S3 Molecular network of ampicillin and ampicillin network features. (a) GNPS molecular network displaying the molecular family in which ampicillin was annotated based on spectral library matching (blue) with unannotated analogs (green). Circles outlined in orange indicate a putative ampicillin analog. Neutral-charge chemical structures proposed are indicated. (b) MS/MS spectra for AMP. Putative fragments ions resulting from fragmentation of the proposed neutral-charge chemical structures are indicated using color. Download FIG S3, TIF file, 2.6 MB. Copyright © 2020 Vrbanac et al. 2020 Vrbanac et al. This content is distributed under the terms of the Creative Commons Attribution 4.0 International license.

10.1128/mSystems.00340-20.4 FIG S4 Ampicillin network features correlate with changes in the metabolome and microbiome. (a) The sum of the peak area of putative ampicillin network features moving down the GI tract; error bars represent the 95% CI. (b) Pearson correlation of ampicillin network features with metabolome pairwise effect size to control (Bray-Curtis) for gut samples (stomach to fecal, down the GI) only from the ampicillin 1-day group. (c) Pearson correlation of ampicillin network features with the change in Shannon diversity (alpha diversity) relative to control samples for gut samples (stomach to fecal, down the GI) only from the AMP.d1 group. The shaded area represents 95% CI. Download FIG S4, TIF file, 1.7 MB. Copyright © 2020 Vrbanac et al. 2020 Vrbanac et al. This content is distributed under the terms of the Creative Commons Attribution 4.0 International license.

In the lower GI tract, differential abundance testing revealed an increase in small di- and tripeptides in AMP.d1 and VAN.d1 mice. To investigate this further, the metabolomics data were processed through PEAKs (23) to yield peptidomics data. For most sections of the lower GI tract and fecal pellet, the total number of peptides was significantly higher in day 1 postantibiotic mice than day 6 postantibiotic mice and untreated controls (Fig. S5A). These peptides aligned to several host proteins, including histones, which were particularly enriched in the AMP.d1 and VAN.d1 mice. Peptide fragments from histones H2A and H2B were significantly elevated in multiple sections of the lower GI (Fig. S5B). These elevated histones could simply be a marker of increased cell death, although histones also play an understudied role in innate immunity (reviewed in reference 24). H2A and H2B can contribute to host defense by direct antimicrobial activity (25, 26) or as components of neutrophil extracellular traps (27). H2A is also elevated in the ileum of chickens with experimental gut damage (28).

10.1128/mSystems.00340-20.5 FIG S5 Small peptides are elevated in the lower GI tract day 1 postantibiotic exposure. (a) Sum of peptide spectral abundances along the lower GI tract comparing AMP.d1- and VAN.d1 (acute antibiotic)-treated mice to control mice and mice at day 6 posttreatment (nonacute). Significance indicates a *P* value of <0.05 by Mann-Whitney U test. (b) Abundances of peptides from histones, both H2A and H2B, were elevated in the lower GI tract of acute antibiotic-treated mice (AMP.d1 and VAN.d1) compared to other groups; significance testing was by dsFDR. Error bars represent the 95% CI. Download FIG S5, TIF file, 1.5 MB. Copyright © 2020 Vrbanac et al. 2020 Vrbanac et al. This content is distributed under the terms of the Creative Commons Attribution 4.0 International license.

To look at microbial and metabolite associations with histones, we ran a random forest regression on colon and cecum samples for both the microbiome and metabolome data, with each body site averaged for each individual mouse, for both histone H2A and histone H2B. Colon and cecum samples were evenly split between the train and test data sets. Regression performance for the microbiome data were modest for H2A (*R*^2^ = 0.59, *P* = 0.0033) and H2B (*R*^2^ = 0.41, *P* = 0.023), and the most important features in the regression model for H2A and H2B were mainly from the *Clostridiales* order and positively associated with histone abundance, except for one *Anaeroplasma* sOTU, the 6th most important feature for H2B, which was negatively correlated with both H2A and H2B abundance. For the metabolomics data, the regression performance was improved for H2A (*R*^2^ = 0.92, *P* = 7.3e−07) and H2B (*R*^2^ = 0.75, *P* = 0.00029), although annotated features were not among the top 20 most important features. The regression was repeated with the metabolome data filtered to only contain putatively annotated features for H2A (*R*^2^ = 0.81, *P* = 0.000068) and H2B (*R*^2^ = 0.737723, *P* = 0.000346). As expected, di- and tripeptides were among the top 10 annotated features for both H2A and H2B. Another top feature for H2A was phytomonic acid, a saturated fatty acid present in bacterial plasma membranes, which was positively associated with histone abundance; accumulation of phytomonic acid is associated with ethanol stress in Oenococcus oeni (29). Furthermore, 9,10-epoxy-12-octadecenoate, a metabolite that neutrophils synthesize during oxidative burst (30), was also among the top features for H2A and positively associated with histone abundance, hinting at neutrophil involvement.

### Metabolome-microbiome interactions.

For gut samples with paired 16S sequencing and metabolomics data, mmvec (31) was used to find cooccurrence probabilities between metabolites and microbes. Metabolites of the same general class, defined by putative GNPS annotations, had similar patterns of cooccurrence with microbes (Fig. S6A). To investigate these relationships in more detail, a multinomial regression model (Songbird [32]) was used to find sOTUs that were highly associated with AMP.d1 and VAN.d1 mice versus controls. *Coprococcus* and *Sutterella* were associated with AMP.d1 mice, and two family S24-7 bacteria were associated with VAN.d1 mice. Looking just at putatively annotated metabolites, all of these sOTUs had high cooccurrence probabilities with tri- or dipeptides and lowest cooccurrence probabilities with 13-docosenamide, a compound equally abundant in blanks (Fig. S6B). *Coprococcus* and *Sutterella*, the sOTUs associated with AMP.d1 mice, had high cooccurrence probabilities with several AMP network features and low cooccurrence probabilities with certain bile acids. Both the S24-7 sOTUs and *Coprococcus* had low cooccurrence probabilities with certain oligosaccharide- or sugar-related molecules, such as maltose. Marker gene sequencing data limits the exploration of the metabolic capacity of these bacteria to produce or consume these metabolites, although PICRUSt2 (33) predictions of functional potential for these two S24-7 and *Coprococcus* sOTUs included maltose O-acetyltransferase (KEGG entry K00661), an enzyme that acetylates maltose and other sugars, and a variety of other sugar transport systems for the *Coprococcus* sOTU (KEGG entries K10112, K10117, K10118, K10119, K03435, and K03436), suggesting the potential for the consumption of sugars. Differential abundance testing recapitulates some of these findings (elevated small peptides and AMP network features), although, in general, we did not observe alterations in the abundance of bile acids or sugar-related molecules. Studies with longer antibiotic courses report altered differential abundance of bile acids and sugars/carbohydrates (34, 35), and our cooccurrence data hint at a relationship between specific microbes enriched with antibiotic treatment and these metabolites.

10.1128/mSystems.00340-20.6 FIG S6 Cooccurrence probabilities for microbes and metabolites cluster by metabolite class. (a) Log conditional cooccurrence probabilities for all microbes and putatively annotated metabolites. The microbial phylum is indicated on the left by row, and general metabolite class is indicated on the top by column (nonbiological metabolites are those that were highly abundant in blanks). (b) A multinomial regression model was used to find sOTUs that were most associated with the AMP.d1 mice (*Coprococcus* and *Sutterella*) and VAN.d1 mice (both family S24-7) compared to the control. Annotated metabolites that had the highest and lowest cooccurrence probabilities with these sOTUs are displayed. Download FIG S6, TIF file, 2.5 MB. Copyright © 2020 Vrbanac et al. 2020 Vrbanac et al. This content is distributed under the terms of the Creative Commons Attribution 4.0 International license.

Administering a single parenteral dose of either of the two common antibiotics did not drastically alter the microbiome or metabolome beyond recovery; in fact, microbiome alterations were primarily resolved by day 6. Nevertheless, a single dose of AMP or VAN still altered the metabolome at day 1 at a variety of body sites, both within and outside the GI tract. Presumably, extended courses of antibiotics, as typically prescribed in multiple daily doses, would exert even greater effects on the global metabolome, as previously seen in studies focused on the GI tract (36). Notably, we observed differential microbiome impacts with antibiotic type and by location in the GI tract. Successful restoration of microbial communities after antibiotic administration may require tailoring for antibiotic type and an understanding of which local GI communities are most impacted.

Organism-wide analyses of the scale we have undertaken remain ambitious and still encumber significant cost and manpower. However, as continual advances in technology have markedly reduced the costs of -omics platform analyses, this work provides a roadmap for understanding the real-time whole-organism-scale biological and chemical effects of antibiotic administration.

### Methods.

**(i) Experimental design.** Thirty-seven-week-old female C57BL/6 mice (Jackson Laboratory) were randomized to 3 or 2 mice per cage upon receipt and allowed to acclimatize for 6 days before antibiotic administration. AMP and VAN were administered by i.p. injection in doses of 100 mg/kg of body weight, dissolved in 100 μl PBS. Control mice were given 100 μl PBS via i.p. injection. Cohoused mice were administered the same antibiotic or PBS alone to avoid coprophagy across treatment groups. All mice, including controls, were dissected at both day 1 and day 6 time points. See Fig. S1 for a graphical representation of experimental design.

**(ii) Sample collection and processing.** Mice were dissected at both the day 1 and day 6 postantibiotic administration time points, alternating between treatment groups. The dissection protocol was as follows. Mice were sacrificed by CO_2_ asphyxiation, followed by cardiac puncture. Dissection was carried out under an open flame with forceps and scissors cleaned with 70% ethanol after the removal of each organ. Fresh fecal samples were collected prior to CO_2_ asphyxiation and organs removed in the following order: spleen, gallbladder, liver, pancreas, stomach, duodenum, jejunum, ileum, cecum, colon, kidneys, adrenal glands, urinary bladder, ovaries, uterine horns, uterine body, cervix, vagina, thymus, heart, lungs, esophagus, trachea, and brain. Organ subsections (e.g., lobes of the liver) were collected in separate tubes for a total of 77 samples per mouse.

Samples were flash frozen in 2-ml Eppendorf tubes in an isopropanol dry ice bath (−77°C) and stored at −80°C. All samples were weighed, and 10 μl of sterile water per milligram of tissue was added to each tube (i.e., 20 mg tissue, 200 μl water). For samples under 15 mg, 150 μl of water was added. Samples were homogenized in a Qiagen TissueLyser II using stainless steel beads.

**(iii) Sample plating scheme.** As the large number of samples required an extensive amount of LC-MS/MS instrument time, which was not available in a continuous block, we separated the samples into three organ groups to avoid issues with comparing treatment groups across metabolomics runs. These groups included “gut” samples (fecal, esophagus, stomach, duodenum, jejunum, ileum, cecum, colon, pancreas, liver, and gallbladder), “reproductive” samples (kidneys, adrenal glands, ovaries, vagina, cervix, uterus, uterine horns, and bladder), and “circulatory” samples (blood, spleen, heart, lung, trachea, thymus, and brain). To avoid plate effects biasing the results, samples were plated by cycling through treatment groups, resulting in every 96-well plate containing samples from multiple treatment groups. Both the metabolome and sequencing plates were prepped at the same time for the gut samples to avoid extra freeze-thaw cycles.

**(iv) Metabolomics preparation and data acquisition.** Five milligrams of tissue homogenate (50 μl) was added to 96-well plates containing 150 μl of 70% high-performance liquid chromatography (HPLC)-grade methanol (chilled) spiked with 5 μM sulfamethoxine (final concentration, ∼50% methanol). Plates were sealed, vortexed, and stored at 4°C overnight (12 h) for extraction. To remove insoluble material, plates were centrifuged at 2,000 rpm at 4°C for 10 min, and 130 μl from each well was aliquoted to a new 96-well plate. Plates were stored at −80°C, and, immediately prior to running on the LC-MS/MS instrument, were centrifuged at 2,000 rpm at 4°C for 10 min; 100 μl was aliquoted to a new 96-well plate. LC-MS/MS data acquisition was performed on a Vanquish ultrahigh-performance liquid chromatography (UPLC) system using a core-shell silica C_18_ column (50 by 2 mm, 1.7-μm particle size, 100-Å pore size; Kinetex, Phenomenex) coupled to a Q Exactive Orbitrap mass spectrometer (Thermo Fisher Scientific, Bremen, Germany). Five microliters of sample was injected and run at 0.5 ml/min on a gradient of solvent A (HPLC-grade water with 0.1% formic acid) and solvent B (HPLC-grade acetonitrile with 0.1% formic acid). The column was maintained at 40°C. The UPLC elution gradient ran for 12.5 min per sample: 5% B from 0 min to 1 min, a linear gradient of 5 to 100% B over 8 min, a hold at 100% B for 2 min, a return to 5% B over 0.5 min, and a hold at 5% B for 2 min to equilibrate the column for the next sample. The flow was directed into a heated electrospray ionization source operated in positive ionization mode with the following parameters: an auxiliary gas flow rate of 14 arbitrary units (a.u.), sweep gas flow rate of 3 a.u., sheath gas flow rate of 52 a.u., spray voltage of +3.5 kV, capillary temperature of 270°C, auxiliary gas heater temperature of 435°C, and S-Lens RF level of 50. The data-dependent acquisition mode was used to acquire the data in which MS1 scans from *m/z* 100 to 1,500 (scan rate, 7 Hz) were followed by an MS2 scan, specifically a product ion scan produced using stepped normalized collision energy higher-energy collisional dissociation, of the five most abundant ions from the prior MS1 scan.

**(v) LC-MS/MS raw data processing.** Raw data were uploaded to MassIVE (https://massive.ucsd.edu/), converted to .mzML files, imported into MZmine2 (37), and truncated at *m/z* 1500 and a 9.5-min retention time (RT). Due to some retention time shift while running the larger gut sample set, parameters are slightly modified versus non-gut sample sets, as indicated in parentheses. The parameters for the gut samples used in MZmine2 are as follows. Mass detection was performed with a noise threshold of 2.0e5 for MS1 and 2.0e3 for MS2 (1.0e3 for non-gut samples) in centroid mode, and chromatograms were built with a 0.05-min time span, 1.0e6 minimum height, and 10-ppm *m/z* tolerance. Chromatograms were deconvoluted with the baseline cutoff algorithm and a minimum peak height of 1.0e6, peak duration of 0.05 to 1.0 min, and baseline of 1.0e4. Isotope peak removal was performed with 15-ppm *m/z* tolerance, 0.3 retention time tolerance (0.05 RT for non-gut samples), and maximum charge of 4, and peaks were aligned with 10-ppm *m/z* tolerance, 75 weight *m/z* tolerance, and 0.4 RT tolerance (0.05 for non-gut samples). Gap filling was also performed with 10% intensity tolerance, 15-ppm *m/z* tolerance, and 0.3 RT tolerance (0.1 for non-gut samples). Peaks were also filtered to remove singletons found in only one sample. Both MS1 and MS2 feature tables were exported, and the “export for GNPS” feature was used to generate a .mgf file for GNPS (18). The signal intensities of the MS1 features were normalized (probabilistic quotient normalization) (38) to the sulfamethoxine internal standard.

**(vi) Metabolite annotation.** GNPS (18) was used to obtain putative annotations for the MS1 feature table via spectral library matching. The following parameters were used: no MS-Cluster, network was filtered to have edges with a cosine score above 0.7 and at least 4 matched peaks, and matches between network spectra and library spectra had a minimum of 6 matched peaks and a minimum cosine score of 0.7. For molecular networking with the raw files, the following parameters were used: MS-Cluster with a parent mass tolerance of 0.02 Da and an MS/MS fragment ion tolerance of 0.02 Da; consensus spectra with fewer than 5 spectra were discarded. The network filtered to have edges with a cosine score above 0.7 and at least 6 matched peaks, and matches between network spectra and library spectra had a minimum cosine score of 0.7 and at least 5 matched peaks.

**(vii) Peptidomics data processing.** LC-MS/MS .mzXML formatted files were loaded into PEAKS Studio 8.51 (23) for the identification of peptidic spectra matching the UniProt mouse protein database (www.uniprot.org; accessed 28 April 2018). Data were imported and refined according to PEAKS settings for Orbitrap instruments under the collision-induced dissociation fragmentation mode. Error tolerance parameters were set to 15-ppm parent mass error tolerance and 0.02-Da fragment mass error tolerance. The search settings included no added restriction enzymes as well as variable modifications for dehydration, acetylation (N terminal), oxidation (M), and formylation (N terminal). The maximum number of variable posttranslational modifications per peptide was set to 3. The label-free quantification mode was used, and the final false discovery rate (FDR) for peptide spectral matches was reported to be 2.9%. Quantification was normalized to the total ion chromatograph.

**(viii) Microbiome sample processing.** DNA was extracted from 100 μl of each gut sample using a MoBio PowerMag soil DNA isolation kit (Qiagen, Carlsbad, CA) by following the Earth Microbiome Project protocol (39). Samples were sequenced on an Illumina MiSeq.

**(ix) Microbiome and metabolome data processing and analysis.** Raw sequencing data were transferred to Qiita (40), where it was demultiplexed, trimmed to 150-bp reads, and denoised to sOTUs using Deblur (41). QIIME2 v2019.4 (42) was used for rarefaction (5,000 sequences per sample), to calculate beta diversity (unweighted UniFrac) and alpha diversity (Shannon) on microbiome data, and to calculate beta diversity (Bray-Curtis) on the metabolome data. Proportions of Gram-negative and Gram-positive bacteria were determined using Bugbase (https://doi.org/10.1101/133462). Bugbase requires an OTU table picked against the GreenGenes database, and closed reference OTU picking was performed with the GreenGenes database (v13_8) (43) at a 97% identity threshold. Using QIIME2 v2019.4 (42), the closed reference OTU table was filtered to exclude blanks and samples with fewer than 5,000 reads. The resulting .biom table was uploaded to BugBase for analysis. For ANCOMv (44) analysis for differentially abundant sOTUs, the nonrarefied feature table was filtered to remove samples with fewer than 5,000 reads/sample and all singleton sOTUs. GI body sites with multiple samples (i.e., colon, cut into 6 sections) were averaged for each mouse prior to performing ANCOM. PICRUSt2 (33) was used to predict functional abundances for sOTUs.

**(x) Statistics and data visualization.** SciPy (45) was used to run the Mann-Whitney U test for comparing proportions of Gram-positive bacteria, delta Shannon distance to control, and peptide sum down the GI tract. SciPy was also used to run Pearson and Spearman correlations. Statsmodels (46) was used to perform *P* value correction for multiple comparisons. As we did not flush the luminal contents, we observed some metabolome variation in GI sections depending on the volume of luminal contents in the section. For differential abundance and beta diversity analyses, these GI sections were averaged per body site per mouse (i.e., colon metabolome was the average of 6 colon sections per mouse). dsFDR (47) was used for differential abundance testing of metabolome data. Beta diversity significance was calculated with the QIIME2 (42) beta-group significance function using PERMANOVA and a Benjamini-Hochberg correction for multiple comparisons. Effect size was calculated by computing the PERMANOVA *R*^2^ values in python, analogous to the r-function of adonis (48). Figures were generated with matplotlib (49) and seaborn (50). The 3D mouse model was generated as described in Quinn et al. (15) from a magnetic resonance image of a mouse. Data visualization of metabolome effect size mapped onto the 3D mouse model was performed with ‘ili (https://ili.embl.de/) (51). Random forest regressions were performed with QIIME2 (42) using 100 trees, 20% of samples were used to test and 80% used to train, and 5 k-fold cross validations were performed.

### Data availability.

Raw data are available on MassIVE under the data sets MSV000082049, MSV000082157, and MSV000082048. Sequencing data are available under EBI accession number ERP121045.
