# Supplementary figures and images for: Evaluating Organism-Wide Changes in the Metabolome and Microbiome following a Single Dose of Antibiotic
Source: mSystems. 2020 Oct 6;5(5):e00340-20. doi: 10.1128/mSystems.00340-20 (PMC7542558; doi:10.1128/mSystems.00340-20)

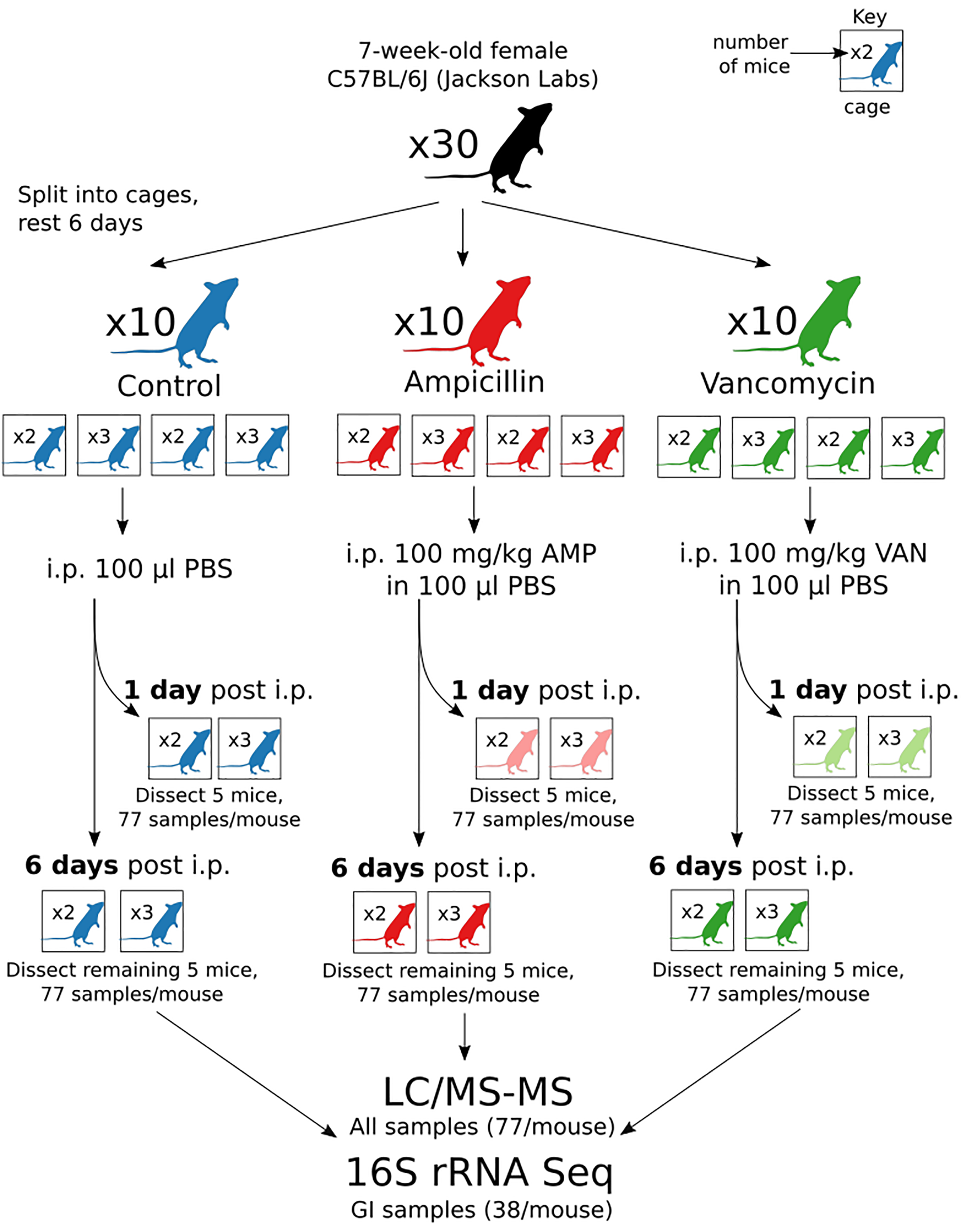

Supplement: FIG S1 [file mSystems.00340-20-sf001.tif]

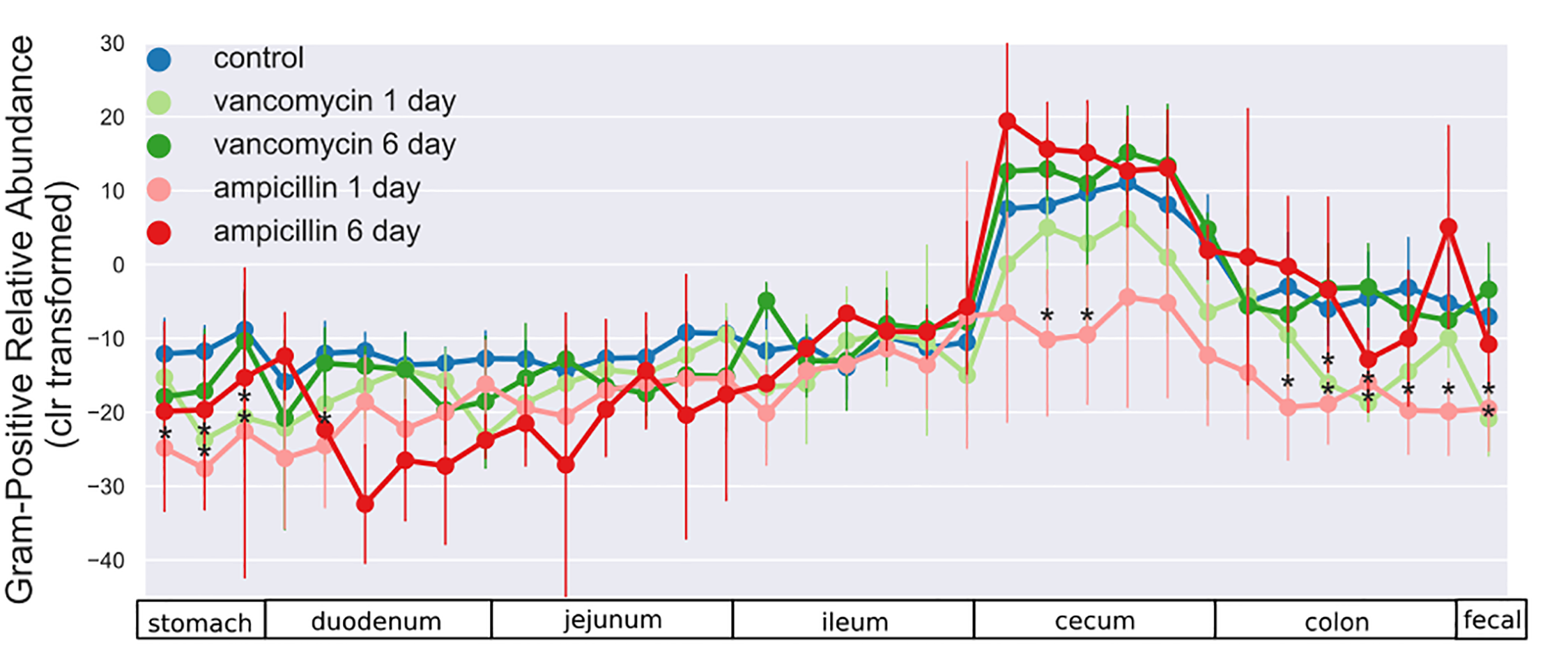

Supplement: FIG S2 [file mSystems.00340-20-sf002.tif]

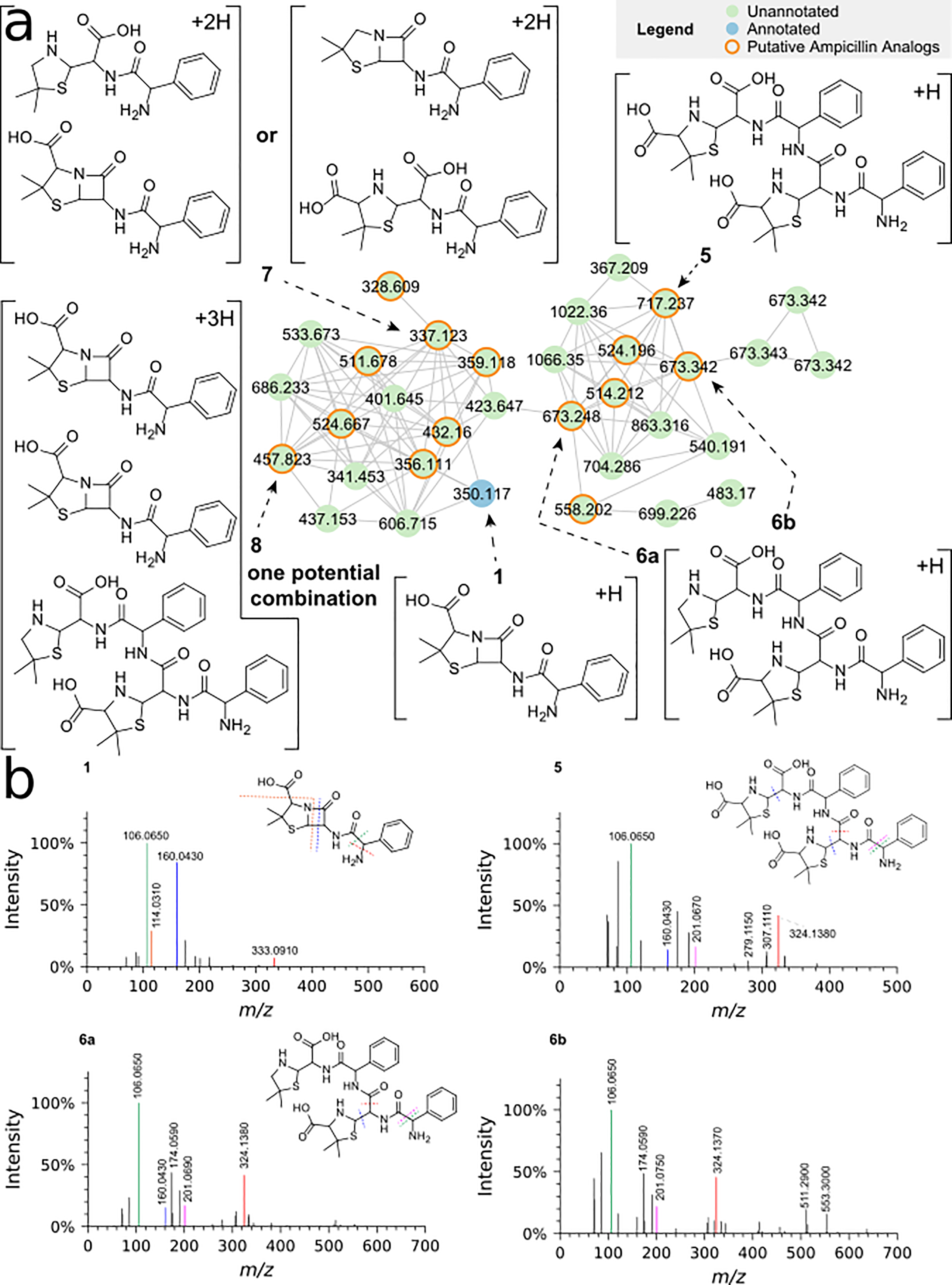

Supplement: FIG S3 [file mSystems.00340-20-sf003.tif]

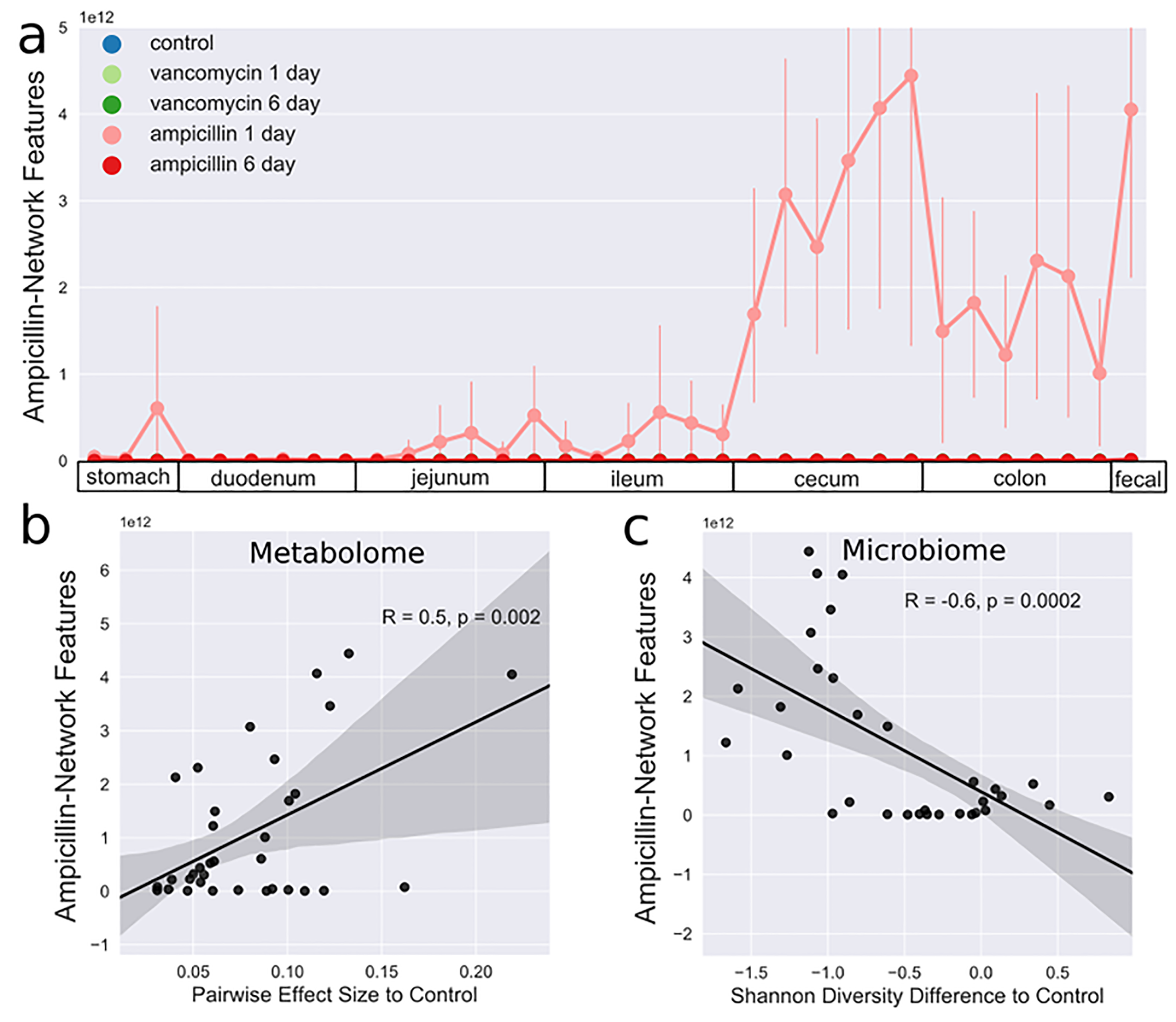

Supplement: FIG S4 [file mSystems.00340-20-sf004.tif]

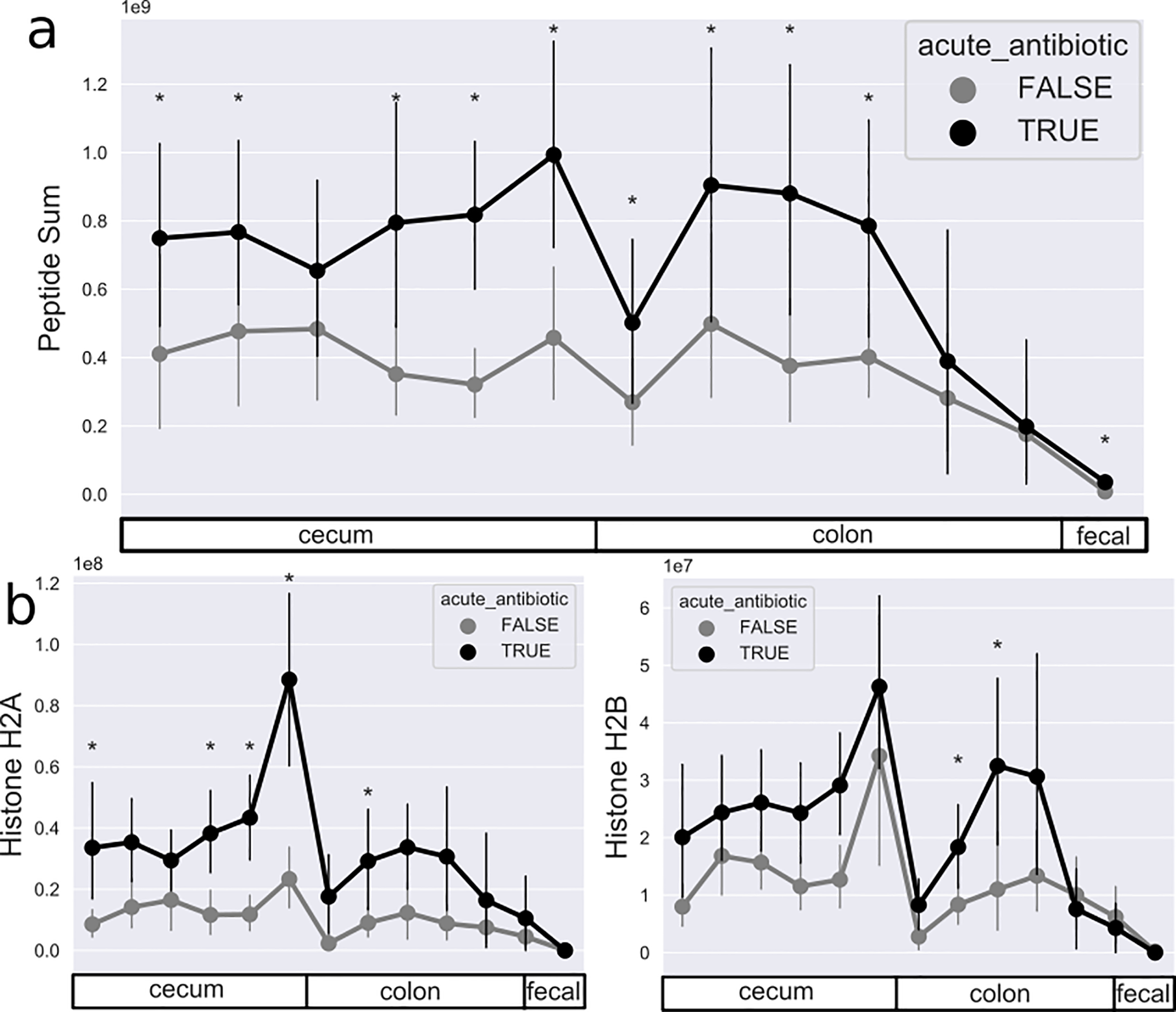

Supplement: FIG S5 [file mSystems.00340-20-sf005.tif]

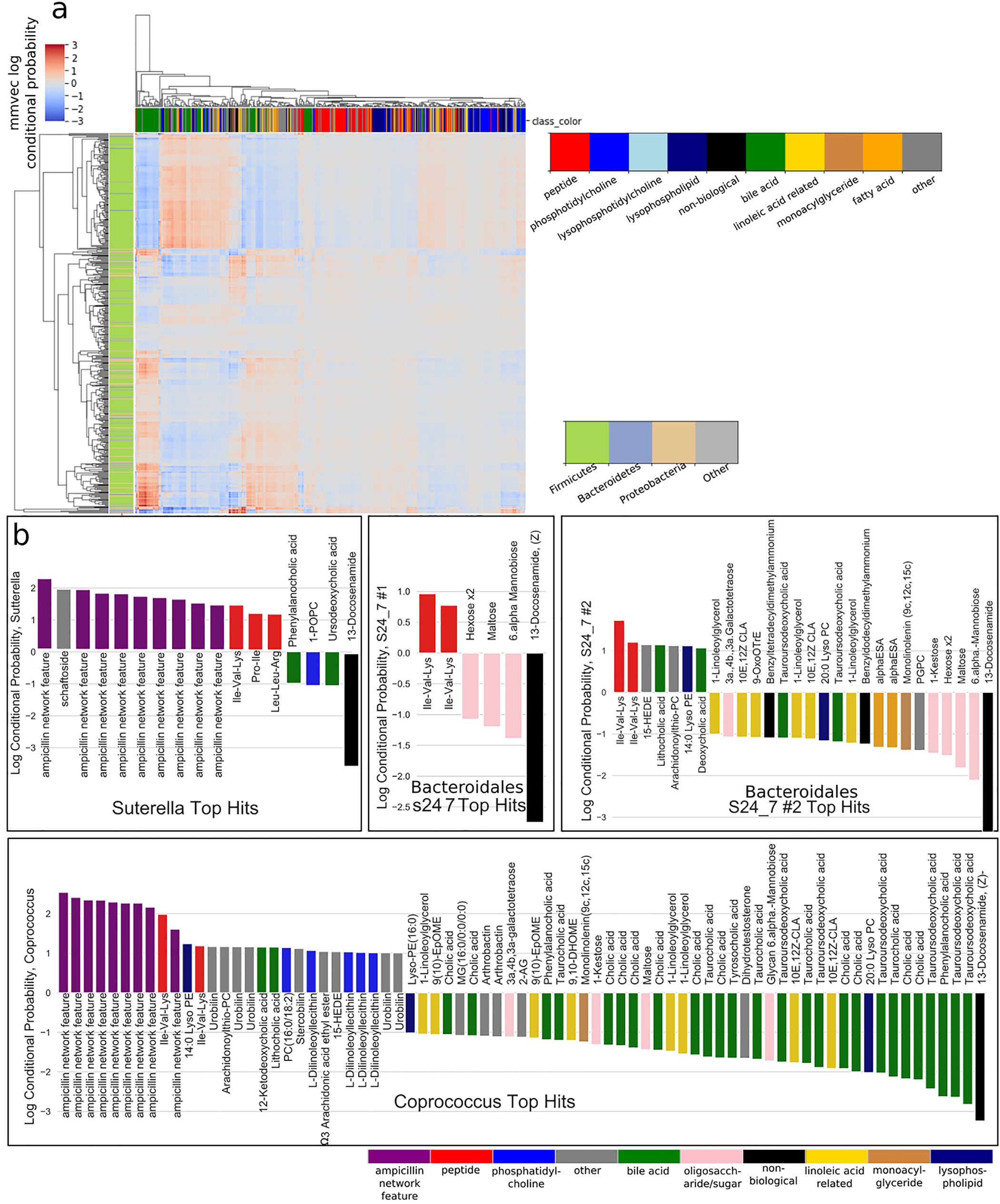

Supplement: FIG S6 [file mSystems.00340-20-sf006.tif]
